# Supplementary material for: MetaRibo-Seq measures translation in microbiomes
Source: Nat Commun. 2020 Jun 29;11:3268. doi: 10.1038/s41467-020-17081-z (PMC7324362; doi:10.1038/s41467-020-17081-z)
Supplement: Supplementary file 10 — Supplementary Data 7 [file 41467_2020_17081_MOESM10_ESM.zip › File2/Confidence_High_Taxonomy/394925_out.krona.html]

Javascript must be enabled to view this page.

members
magnitude
magnitudeUnassigned
count
unassigned
taxon
rank

394925\_out

411

superkingdom
411
2

411
976
phylum

class
200643
411

411
171549
order

family
1
1853231

genus
1
283168


SRS017521\_contig\_number\_49627
28118
1
species

2005525
2
family

375288
2
genus

species
46503
1

SRS149325\_contig\_number\_15657


SRS011084\_contig\_number\_contig-100\_253.179252
1
387661
species

family
171550
1

239759
1
genus

species
1
1896968

SRS049402\_contig\_number\_9510

family
815
406

genus

SRS012849\_contig\_number\_15894SRS015264\_contig\_number\_18864SRS018427\_contig\_number\_31416SRS023583\_contig\_number\_11458SRS056519\_contig\_number\_2491SRS058145\_contig\_number\_3620SRS076976\_contig\_number\_17814SRS077086\_contig\_number\_8038SRS098717\_contig\_number\_21205SRS148319\_contig\_number\_contig-100\_1036.93673SRS893230\_contig\_number\_11113
406
816
11


SRS148721\_contig\_number\_55269
species
1
28111


SRS046717\_contig\_number\_7455
species
1
585543

1
2292284
species

SRS024132\_contig\_number\_45268

species
2292927
1

SRS023715\_contig\_number\_23566

species
33
357276

SRS013215\_contig\_number\_173SRS013940\_contig\_number\_12259SRS015794\_contig\_number\_13080SRS015890\_contig\_number\_22896SRS016018\_contig\_number\_6518SRS018049\_contig\_number\_50SRS018541\_contig\_number\_4290SRS018575\_contig\_number\_5475SRS018984\_contig\_number\_15905SRS020869\_contig\_number\_23803SRS024435\_contig\_number\_19515SRS042628\_contig\_number\_36162SRS043001\_contig\_number\_10067SRS045713\_contig\_number\_3409SRS048981\_contig\_number\_2962SRS052078\_contig\_number\_110SRS054590\_contig\_number\_887SRS057717\_contig\_number\_5618SRS058070\_contig\_number\_26303SRS058770\_contig\_number\_14018SRS075341\_contig\_number\_15270SRS077024\_contig\_number\_9766SRS098655\_contig\_number\_3446SRS101268\_contig\_number\_261SRS1041038\_contig\_number\_16956SRS1041112\_contig\_number\_2514SRS1055034\_contig\_number\_8061SRS142712\_contig\_number\_1784SRS143181\_contig\_number\_13768SRS143598\_contig\_number\_5662SRS148091\_contig\_number\_12387SRS149784\_contig\_number\_24806SRS149879\_contig\_number\_24096


SRS014313\_contig\_number\_185SRS014613\_contig\_number\_367SRS042284\_contig\_number\_4577
species
3
47678

species
1262741
1

SRS013800\_contig\_number\_8518


SRS015264\_contig\_number\_12430SRS023346\_contig\_number\_83SRS045195\_contig\_number\_7913SRS048060\_contig\_number\_12432SRS050299\_contig\_number\_7057SRS055982\_contig\_number\_3558SRS064232\_contig\_number\_4276SRS064276\_contig\_number\_27280SRS077392\_contig\_number\_41948SRS078419\_contig\_number\_11756SRS101433\_contig\_number\_14714SRS1041145\_contig\_number\_29919SRS1054691\_contig\_number\_15142SRS1055043\_contig\_number\_9779SRS1055067\_contig\_number\_4949SRS1055099\_contig\_number\_14585SRS148874\_contig\_number\_12028
species
17
2292935

species
2292944
3

SRS018427\_contig\_number\_27290SRS062701\_contig\_number\_134SRS1041157\_contig\_number\_280


SRS050941\_contig\_number\_13375SRS063813\_contig\_number\_1417SRS077231\_contig\_number\_19587SRS078242\_contig\_number\_20940SRS098644\_contig\_number\_15186SRS103987\_contig\_number\_25751SRS1054716\_contig\_number\_1907SRS1055069\_contig\_number\_163SRS143070\_contig\_number\_2126SRS143991\_contig\_number\_27313SRS145497\_contig\_number\_1702
11
46506
species


SRS017821\_contig\_number\_11986
1
1638780
species


SRS077127\_contig\_number\_3709
1
1638779
species

species
1
1262743

SRS013951\_contig\_number\_37418

species
2
2292941

SRS1041147\_contig\_number\_238SRS893369\_contig\_number\_2712

species
1
371601

SRS148319\_contig\_number\_349

species
821
288

SRS011134\_contig\_number\_50743SRS011239\_contig\_number\_14369SRS011302\_contig\_number\_28030SRS011405\_contig\_number\_17452SRS012969\_contig\_number\_30977SRS013098\_contig\_number\_12043SRS013158\_contig\_number\_4122SRS013382\_contig\_number\_71SRS013521\_contig\_number\_4754SRS013638\_contig\_number\_16717SRS013965\_contig\_number\_35031SRS014235\_contig\_number\_703SRS014287\_contig\_number\_18836SRS014412\_contig\_number\_5174SRS014459\_contig\_number\_contig-100\_2498.200582SRS014534\_contig\_number\_528SRS014683\_contig\_number\_14285SRS014736\_contig\_number\_2700SRS014855\_contig\_number\_910SRS014923\_contig\_number\_45950SRS014948\_contig\_number\_20906SRS015065\_contig\_number\_contig-100\_2280.187043SRS015190\_contig\_number\_14152SRS015217\_contig\_number\_22837SRS015431\_contig\_number\_51029SRS015486\_contig\_number\_407SRS015782\_contig\_number\_25682SRS015854\_contig\_number\_contig-100\_162.113150SRS015960\_contig\_number\_18465SRS016056\_contig\_number\_5380SRS016095\_contig\_number\_2210SRS016132\_contig\_number\_163SRS016203\_contig\_number\_contig-100\_1486.75908SRS016335\_contig\_number\_contig-100\_262.216452SRS016629\_contig\_number\_766SRS016753\_contig\_number\_7316SRS016840\_contig\_number\_832SRS016897\_contig\_number\_406SRS016954\_contig\_number\_15249SRS016989\_contig\_number\_12295SRS017103\_contig\_number\_1719SRS017191\_contig\_number\_17041SRS017247\_contig\_number\_5425SRS017375\_contig\_number\_53SRS017433\_contig\_number\_10305SRS017701\_contig\_number\_3126SRS017745\_contig\_number\_459SRS017859\_contig\_number\_3574SRS018133\_contig\_number\_1457SRS018351\_contig\_number\_11083SRS018623\_contig\_number\_1271SRS018817\_contig\_number\_10557SRS018836\_contig\_number\_14461SRS018888\_contig\_number\_2388SRS018936\_contig\_number\_6584SRS019030\_contig\_number\_2126SRS019068\_contig\_number\_41530SRS019178\_contig\_number\_317SRS019267\_contig\_number\_6908SRS019381\_contig\_number\_11481SRS019445\_contig\_number\_27016SRS019496\_contig\_number\_646SRS019601\_contig\_number\_29458SRS019693\_contig\_number\_2253SRS019808\_contig\_number\_17910SRS019968\_contig\_number\_10149SRS020233\_contig\_number\_26156SRS020328\_contig\_number\_28665SRS020394\_contig\_number\_126SRS020508\_contig\_number\_149SRS020622\_contig\_number\_729SRS021153\_contig\_number\_3725SRS021219\_contig\_number\_8646SRS021484\_contig\_number\_27684SRS021948\_contig\_number\_1498SRS022071\_contig\_number\_25289SRS022137\_contig\_number\_13392SRS022609\_contig\_number\_41674SRS022713\_contig\_number\_2022SRS023526\_contig\_number\_28648SRS023829\_contig\_number\_131SRS023914\_contig\_number\_27500SRS023971\_contig\_number\_118SRS024075\_contig\_number\_3273SRS024492\_contig\_number\_12372SRS042966\_contig\_number\_16406SRS043411\_contig\_number\_730SRS043667\_contig\_number\_218SRS043701\_contig\_number\_11186SRS043768\_contig\_number\_9872SRS044535\_contig\_number\_384SRS045004\_contig\_number\_10154SRS045244\_contig\_number\_1084SRS045645\_contig\_number\_16608SRS045739\_contig\_number\_11241SRS045826\_contig\_number\_5259SRS046068\_contig\_number\_1500SRS046369\_contig\_number\_13508SRS046502\_contig\_number\_10430SRS046810\_contig\_number\_contig-100\_31.32SRS047014\_contig\_number\_contig-100\_326.161377SRS047044\_contig\_number\_14086SRS047433\_contig\_number\_18825SRS048164\_contig\_number\_30271SRS049446\_contig\_number\_134SRS049712\_contig\_number\_contig-100\_426.130200SRS049773\_contig\_number\_8553SRS049896\_contig\_number\_30085SRS049959\_contig\_number\_contig-100\_393.274059SRS049995\_contig\_number\_10437SRS050026\_contig\_number\_2554SRS050422\_contig\_number\_36471SRS050520\_contig\_number\_11SRS050752\_contig\_number\_contig-100\_984.159794SRS050801\_contig\_number\_2476SRS050974\_contig\_number\_1827SRS050998\_contig\_number\_13600SRS051882\_contig\_number\_9734SRS052576\_contig\_number\_22SRS052697\_contig\_number\_40539SRS053087\_contig\_number\_contig-100\_584.38411SRS053214\_contig\_number\_18610SRS053335\_contig\_number\_9732SRS053356\_contig\_number\_47045SRS053398\_contig\_number\_67SRS054059\_contig\_number\_4418SRS054352\_contig\_number\_3129SRS054905\_contig\_number\_1040SRS055017\_contig\_number\_34551SRS055966\_contig\_number\_110SRS056273\_contig\_number\_20897SRS057478\_contig\_number\_2138SRS057480\_contig\_number\_9000SRS058268\_contig\_number\_27SRS058723\_contig\_number\_1997SRS062427\_contig\_number\_7572SRS062654\_contig\_number\_2229SRS063127\_contig\_number\_600SRS063190\_contig\_number\_2403SRS063370\_contig\_number\_4156SRS063489\_contig\_number\_120SRS063518\_contig\_number\_23268SRS063928\_contig\_number\_547SRS064645\_contig\_number\_575SRS064973\_contig\_number\_16SRS065176\_contig\_number\_14374SRS065397\_contig\_number\_3930SRS074586\_contig\_number\_4105SRS074670\_contig\_number\_277SRS074964\_contig\_number\_5572SRS075716\_contig\_number\_133SRS075821\_contig\_number\_117SRS075878\_contig\_number\_23790SRS075984\_contig\_number\_17309SRS076756\_contig\_number\_15935SRS076876\_contig\_number\_426SRS077194\_contig\_number\_6245SRS077294\_contig\_number\_11522SRS077335\_contig\_number\_7996SRS077454\_contig\_number\_3064SRS077502\_contig\_number\_494SRS077552\_contig\_number\_3843SRS077641\_contig\_number\_3788SRS077849\_contig\_number\_23684SRS077991\_contig\_number\_contig-100\_329.330SRS078176\_contig\_number\_4478SRS078665\_contig\_number\_6692SRS097889\_contig\_number\_17129SRS097920\_contig\_number\_25553SRS097958\_contig\_number\_7803SRS098061\_contig\_number\_8277SRS098073\_contig\_number\_11919SRS098571\_contig\_number\_9887SRS098827\_contig\_number\_1007SRS098881\_contig\_number\_25329SRS100021\_contig\_number\_1250SRS100907\_contig\_number\_8022SRS101376\_contig\_number\_14477SRS101448\_contig\_number\_contig-100\_1483.1483SRS104084\_contig\_number\_3251SRS1041031\_contig\_number\_8793SRS1041032\_contig\_number\_16622SRS1041036\_contig\_number\_1953SRS1041037\_contig\_number\_14739SRS1041091\_contig\_number\_17118SRS1041092\_contig\_number\_10716SRS1041116\_contig\_number\_8751SRS1041118\_contig\_number\_contig-100\_413.95604SRS1041129\_contig\_number\_15057SRS1041130\_contig\_number\_241SRS1041133\_contig\_number\_28382SRS1041134\_contig\_number\_10766SRS1041136\_contig\_number\_15706SRS1041137\_contig\_number\_13139SRS1041138\_contig\_number\_9935SRS1041140\_contig\_number\_18603SRS1041141\_contig\_number\_196SRS1041142\_contig\_number\_739SRS1041144\_contig\_number\_8219SRS1041164\_contig\_number\_5183SRS104327\_contig\_number\_2885SRS104400\_contig\_number\_18461SRS104485\_contig\_number\_19SRS104693\_contig\_number\_3679SRS104912\_contig\_number\_13306SRS104912\_contig\_number\_contig-100\_584.113173SRS104975\_contig\_number\_18255SRS104975\_contig\_number\_22781SRS105082\_contig\_number\_189SRS1054928\_contig\_number\_contig-100\_1743.43579SRS1055038\_contig\_number\_4968SRS1055049\_contig\_number\_10930SRS1055056\_contig\_number\_12995SRS1055076\_contig\_number\_14294SRS1055095\_contig\_number\_709SRS140513\_contig\_number\_15458SRS142503\_contig\_number\_28838SRS142542\_contig\_number\_20136SRS142599\_contig\_number\_23622SRS142890\_contig\_number\_contig-100\_2323.207366SRS142923\_contig\_number\_13709SRS142980\_contig\_number\_2677SRS143085\_contig\_number\_706SRS143148\_contig\_number\_22104SRS143181\_contig\_number\_10494SRS143342\_contig\_number\_13428SRS143372\_contig\_number\_236SRS143417\_contig\_number\_29184SRS143523\_contig\_number\_16255SRS143780\_contig\_number\_3853SRS143876\_contig\_number\_9655SRS143895\_contig\_number\_19525SRS144183\_contig\_number\_41859SRS144297\_contig\_number\_11533SRS144362\_contig\_number\_6436SRS144506\_contig\_number\_41772SRS144537\_contig\_number\_4098SRS144603\_contig\_number\_11372SRS144714\_contig\_number\_25067SRS144753\_contig\_number\_11734SRS145308\_contig\_number\_1852SRS146764\_contig\_number\_4032SRS146813\_contig\_number\_15172SRS147022\_contig\_number\_3607SRS147039\_contig\_number\_1960SRS147346\_contig\_number\_4792SRS147377\_contig\_number\_9131SRS147425\_contig\_number\_554SRS147557\_contig\_number\_27796SRS147614\_contig\_number\_2396SRS147653\_contig\_number\_7554SRS147766\_contig\_number\_2832SRS147977\_contig\_number\_1251SRS148091\_contig\_number\_9239SRS148196\_contig\_number\_38297SRS148253\_contig\_number\_2708SRS148424\_contig\_number\_32533SRS148511\_contig\_number\_4680SRS148784\_contig\_number\_contig-100\_1983.158511SRS148817\_contig\_number\_3745SRS148970\_contig\_number\_120SRS149075\_contig\_number\_10332SRS149181\_contig\_number\_13531SRS149244\_contig\_number\_10730SRS150029\_contig\_number\_13786SRS892882\_contig\_number\_11077SRS893170\_contig\_number\_7852SRS893172\_contig\_number\_9164SRS893187\_contig\_number\_17243SRS893252\_contig\_number\_11771SRS893270\_contig\_number\_23322SRS893279\_contig\_number\_115SRS893285\_contig\_number\_532SRS893288\_contig\_number\_3858SRS893292\_contig\_number\_1660SRS893292\_contig\_number\_9593SRS893295\_contig\_number\_13153SRS893300\_contig\_number\_6715SRS893327\_contig\_number\_contig-100\_5214.56806SRS893342\_contig\_number\_7896SRS893366\_contig\_number\_22322SRS893373\_contig\_number\_20771SRS893378\_contig\_number\_21202SRS893380\_contig\_number\_2558SRS893382\_contig\_number\_5565SRS893383\_contig\_number\_1628SRS971275\_contig\_number\_32203SRS971276\_contig\_number\_31553

2
28116
species

SRS140645\_contig\_number\_1389SRS893358\_contig\_number\_4307

species
5
671267

SRS013476\_contig\_number\_33626SRS016381\_contig\_number\_contig-100\_227.35065SRS024388\_contig\_number\_273SRS1041132\_contig\_number\_195SRS147139\_contig\_number\_10823

3
818
species

SRS015694\_contig\_number\_560SRS140492\_contig\_number\_22111SRS893253\_contig\_number\_29


SRS012273\_contig\_number\_2073
species
2292002
1

1
310297
species

SRS077589\_contig\_number\_627


SRS143722\_contig\_number\_11143
species
1
291644


SRS075773\_contig\_number\_11
1
246787
species

species
1
457391

SRS075021\_contig\_number\_18


SRS012902\_contig\_number\_106SRS015578\_contig\_number\_20898SRS016495\_contig\_number\_14693SRS019397\_contig\_number\_183SRS024549\_contig\_number\_2589SRS024663\_contig\_number\_6813SRS063985\_contig\_number\_16403SRS075398\_contig\_number\_548SRS077730\_contig\_number\_189
species
817
9

species
204516
1

SRS052027\_contig\_number\_10869

2
2292943
species

SRS019286\_contig\_number\_578SRS104636\_contig\_number\_20946


SRS015663\_contig\_number\_26177SRS1055083\_contig\_number\_2903
species
2
1263040

1
171552
family

1
577309
genus


SRS017307\_contig\_number\_12599
species
1
454154
